# Depressive Symptom Severity and Community Collective Efficacy following the 2004 Florida Hurricanes

**DOI:** 10.1371/journal.pone.0130863

**Published:** 2015-06-30

**Authors:** Carol S. Fullerton, Robert J. Ursano, Xian Liu, Jodi B. A. McKibben, Leming Wang, Dori B. Reissman

**Affiliations:** 1 Department of Psychiatry, Uniformed Services University of the Health Sciences, Bethesda, Maryland, United States of America; 2 Department of Psychology, West Chester University, West Chester, Pennsylvania, United States of America; Univ of Toledo, UNITED STATES

## Abstract

There is a lack of research investigating community-level characteristics, such as community collective efficacy, mitigating the impact of disasters on psychological health, specifically depression. We examined the association of community collective efficacy with depressive symptom severity in Florida public health workers (n = 2249) exposed to the 2004 hurricane season using a multilevel approach. Cross-sectional anonymous questionnaires were distributed electronically to all Florida Department of Health (FDOH) personnel that assessed depressive symptom severity and collective efficacy nine months after the 2004 hurricane season. Analyses were conducted at the individual level and community level using zip codes. The majority of participants were female (81.9%), and ages ranged from 20 to 78 years (median = 49 years). The majority of participants (73.4%) were European American, 12.7% were African American, and 9.2% were Hispanic. Using multilevel analysis, our data indicate that higher community-level and individual-level collective efficacy were associated with significantly lower depressive symptom severity (b = -0.09 [95% CI: -0.13, -0.04] and b = -0.09 [95% CI: -0.12, -0.06], respectively) even after adjusting for individual sociodemographic variables, community socioeconomic characteristics, individual injury/damage, and community storm damage. Lower levels of depressive symptom severity were associated with communities with high collective efficacy. Our study highlights the possible importance of programs that enrich community collective efficacy for disaster communities.

## Introduction

State and local public health workers play a critical role as first responders. Concern over public health response to natural disasters has increased in the aftermath of the 2004 Asian tsunami, Hurricanes Katrina and Rita, and the 2010 earthquakes in Haiti and Chile. Public health workers living in disaster-affected communities experience the direct effect of disasters, and at the same time are responsible for providing care to others. Public health workers include individuals who provide medical care as well as those in traditional state public health positions, such as epidemiologists and laboratory technicians. Public health workers exposed to disasters have high rates of disaster-related distress and posttraumatic stress disorder (PTSD) [1–9], as well as other psychiatric disorders including depression and alcohol abuse following a disaster [10–13]. Symptoms of major depression were reported by 27% of firefighters 13 weeks after responding to Hurricane Katrina [12], and importantly, symptoms of depression increase with the number of critical work-related exposures in first responders [11] Although depression is often co-morbid with PTSD, it is not always present, and has different risk and protective factors and a different course than PTSD, suggesting the importance of examining depression following trauma.

Multiple community characteristics influence mental health outcomes [8, 14, 15]. The majority of studies of disaster mental health, which address neighborhood and social processes, measure and analyze them as individual-level variables [16, 17]. Collective efficacy, defined as social cohesion among neighbors and their willingness to intervene for the common good [14], can be both an individual-level perception and a community-level capacity. Individual perception of collective efficacy refers to the strength of one’s community in two domains, informal social control and social cohesion/trust in neighbors [14]. Community collective efficacy is a measure of the community members’ average perception of collective efficacy associated with their community. At the community level, willingness to intervene for the common good depends on mutual trust and solidarity among neighbors.

Nearly all studies supported the association of neighborhood characteristics and depressive symptoms in a review of studies of neighborhoods and depression [18]. Increases in community collective efficacy are associated with lower levels of depressive symptoms [18, 19] and may moderate the effects of stressors, such as perceived discrimination, on depression [20]. The protective effects of community collective efficacy on depression may be due, in part, to community resources that buffer psychological responses to stressful events [19, 21, 22], and promote conservation of individual resources [23]. In contrast, exposure to both displacement and low social cohesion following Hurricane Katrina was associated with higher risk for depression [24], highlighting the importance of both the individual- and community-level social environment in psychological functioning. Similarly, perception of neighborhood-level income inequality (characterized by poor quality or lack of resources) was related to increased depression following 9/11, particularly in residents with lower income, who may be more vulnerable to the effects of disasters, dependent on community resources, and experience lower social cohesion [25].

In the United States, the 2004 Florida hurricane season was unprecedented. Four hurricanes (Charley, Frances, Ivan, and Jeanne) and one tropical storm (Bonnie) made landfall within a period of seven weeks [26]. Nearly $4 billion in recovery costs was provided to individuals and communities [27]. Our previous study of Florida Department of Health (FDOH) public health workers following the 2004 hurricanes has shown a high mental and behavioral health burden on the public health workers of the disaster [4, 7]. In addition, higher levels of individual and community-level collective efficacy were associated with lower rates of PTSD [8]. Given the differences between depression and PTSD, and previous studies indicating the association of depression to individual and community collective efficacy, it is important to better understand the association of depression and collective efficacy following disaster. Such studies can identify prevention and intervention strategies specific to depression for public health workers.

In previous studies using the current sample and survey instrument, we have shown that, (1) PTSD, depression, alcohol and tobacco use, and sleep and arousal are important mental health and behavioral outcomes of this disaster [4,7], and (2) collective efficacy is associated with PTSD outcomes [8]. In the current study, using the same sample and survey, we examine the association of collective efficacy to depression. Depression is a distinct disorder associated with disasters with different symptoms and different treatment implications than PTSD. Understanding the relationship of collective efficacy to depression can suggest new possible public health planning surveillance and intervention opportunities.

The present study examines the relationship of both community level and individual level collective efficacy, as well as injury/damage to depressive symptoms in the same population of FDOH public health workers nine months after the 2004 hurricanes.

## Methods

### Ethics Statement

The study was conducted in accordance with the ethical standards and approval of the Institutional Review Board, Uniformed Services University of the Health Sciences, Bethesda, MD. Participation was voluntary. Approximately nine months after the 2004 hurricane season questionnaires and a project description were distributed to FDOH employees using the personnel e-mail distribution lists. All participants indicated agreement to participate by completing a questionnaire that was transmitted electronically and anonymously.

### Participants and Procedures

This study reports on the association of depression and collective efficacy using the same sample and survey previously published by the authors where we previously examined core outcomes, sleep, PTSD and collective efficacy [4,7,8]. Two versions of the questionnaire (i.e., A and B) were distributed randomly so that each potential participant received either version. Questionnaire versions contained some of the same items and some unique items, with version A focusing on mental health items. Of an estimated 8564 FDOH personnel who worked during the 2004 hurricanes and were available at the time of the survey, we were able to contact and invite 6637 individuals to participate. After reading a description of the study and the informed consent, 4323 agreed to participate and completed and returned the questionnaire (Version A = 2249; Version B = 2074), for an estimated response rate of 65.1%. This study included respondents who completed Version A. Ages of the participants ranged from 20 to 78 years (median = 49 years). The majority were female (80.4%; n = 1787) and currently married (65.6%, n = 1242). Also, the majority were White (73.4%, n = 1390), 12.7% (n = 240) were African American, 9.2% (n = 175) were Hispanic, and 4.7% (n = 88) other. Approximately half of the participants had less than a BA/BS degree (50.4%, n = 954). Prior trauma exposure only as a child was reported by 5.7% (n = 128) of participants, 20.8% (n = 464) reported prior trauma exposure only as an adult, and 14.8% (n = 330) reported prior trauma exposure both as an adult and as a child. Occupations of the sample were: administration/program management (54%, n = 1188); medical (27%, n = 588); epidemiologist/outbreak investigator (7%, n = 145); statistical/IT services (6%, n = 137); and support services/maintenance (6%, n = 105).

### Measures

#### Depression

Depressive symptom severity scores were assessed with the nine-item Patient Health Questionnaire Depression Scale-9 (PHQ-9) [28, 29]. The PHQ-9 lists all symptoms of DSM-IV Criterion A of Major Depressive Episode [30]. Respondents rated how much they had been bothered by each symptom in the previous two weeks on a scale ranging from 0, not at all to 3, nearly every day. Responses are summed to produce a depressive symptom severity score ranging from 0 to 27.

#### Collective Efficacy

Collective efficacy was assessed with the 10-item scale employed by Sampson and colleagues [14]. The scale has five items in each of two domains: informal social control and social cohesion / trust scored on five-point Likert scales (ranging from very likely to very unlikely, and strongly disagree to strongly agree, respectively), and summed to produce a total score for individual level collective efficacy ranging from 10 to 50. Informal social control includes five items that ask how likely it would be that respondents’ neighbors could be counted on to intervene if: a) children were skipping school and hanging out on a street corner; b) children were spray painting graffiti on a local building; c) children were showing disrespect to an adult; d) a fire broke out in front of their house; and e) if a fire station closest to their home was threatened with budget cuts. The social cohesion / trust scale has five items that assess the extent to which participants agreed that in their home neighborhood: a) people are willing to help their neighbors; b) it is a close-knit neighborhood; c) people can be trusted; d) people generally get along with each other; and e) people share the same values. Higher scores indicate greater collective efficacy.

Community level collective efficacy was assessed using zip codes to define the community units. For each zip code, the sample mean of those individuals in the zip code was obtained and rescaled as a centered variable about the grand mean of the entire sample. Since a zip code represents a collection of people and institutions that occupy a unique subsection of a geographic location, each zip code is sufficiently externally heterogeneous and internally homogeneous to be used in multilevel analyses. Given this design, 825 zip codes served as the level-two unit in this study. Sampson and colleagues [14] demonstrated high between-neighborhood reliability (ranging from 0.80 to 0.91) across 343 neighborhoods in Chicago, IL. There was a strong association between social cohesion and informal social control across neighborhoods (r = 0.80, p < 0001), suggesting that these scales were measuring aspects of the same latent construct.

#### Individual hurricane injury/damage

Injury/damage at the time of the hurricanes was assessed as an individual-level variable by asking participants whether they had experienced any of the following six events during each of the five hurricanes: loss of electrical power; damage to vehicle; injury or harm to self; injury or harm to spouse/significant other; injury/harm to children; and injury/harm to pets. Those reporting at least two of the events during the five hurricanes were considered to have high hurricane-related injury/damage (n = 1093, 58.14%).

#### Community hurricane damage

Using FEMA county data for all five storms [27], we identified the zip code level of FEMA public and individual assistance received. Each zip code was scored based on its highest community storm damage across the five storms to index the level of individual and public assistance received. The level of community storm damage was assessed using FEMA categories A to G (A. debris removal, B. emergency protective measures, C. roads and bridges, D. water control facilities, E. public buildings and equipment, F. public utilities, and G. recreational or other) [31]. We combined categories to create five levels of public assistance and, therefore, community damage, as follows: 0 = no assistance; 1 = individual assistance only; 2 = public assistance for category B; 3 = public assistance for categories A-B; and 4 = public assistance for categories A-G. This level-two variable was then centered.

#### Socioeconomic characteristics

Ten zip code-specific census measures assessed socioeconomic characteristics [8]. Following Sampson’s model [14], three community-level factor scores, concentrated disadvantage, immigrant concentration, and residential stability, were extracted from the ten zip code-specific census measures. We used a principal factor analysis with squared multiple correlations (SMC) for the prior communality estimates. Both orthogonal and oblique rotations were applied. The oblique rotated factor pattern was highly consistent with those reported by Sampson and associates [8, 14]. Factor 1, concentrated disadvantage, had an eigenvalue of 3.94, with high loadings for poverty, receipt of public assistance, unemployment, female-headed families, density of children, percentage of Black residents, and percentage of owner-occupied homes. Factor 2, immigrant concentration, captured two variables with high loadings for percentage of Latinos and percentage of foreign-born individuals. Factor 3, residential stability, had one variable with a high loading for percentage of persons living in the same house for the past five years. The three factors were constructed as standardized scores with a mean of 0 and a standard deviation of 1. These factors were used as level-2 control variables in the multilevel analyses.

### Statistical Analysis

Potential individual and community-level risk factors for higher depressive symptom severity scores at 9 months post-hurricane in FDOH employees were analyzed using a multilevel modeling approach. The level 1 unit was individuals (n = 1893) and the level-2 unit was zip code-defined communities (n = 825). Mean collective efficacy was calculated using all available data (N = 2.249) yielding a mean of 2.73 participants in each zip code for this calculation. All subsequent analyses excluded missing cases across all covariates (n = 1893). Statistical analyses were conducted using SAS software Version 9.2 [32]. Specifically, we applied SAS PROC MIXED procedure that uses empirical Bayesian approach for handling low reliability in some of the level-2 units [33, 34].

Random coefficient analyses were used to evaluate the associations with depressive symptoms. The individual-level collective efficacy predictor was considered in the presence of individual and community-level control variables. The interaction between injury/damage and individual-level collective efficacy, and the interaction between injury/damage and community storm damage were included as additional fixed effects. We considered three random effects for the intercept, for the slopes of injury/damage, and for the participants within communities. The degree of clustering within zip codes was assessed by the intra-communities correlation [35]. We applied the same multilevel approach for community-level collective efficacy, except that individual collective efficacy was replaced by community-level collective efficacy. We constructed a multilevel model by including all of the aforementioned covariates.

## Results

Nine months after the 2004 hurricanes, high levels of individual injury/damage and high levels of community storm damage were reported in this group of FDOH workers. Specifically, 57.95% (n = 1097) experienced high levels of personal injury/damage, and the average level of community storm damage was 1.50 (SD = 1.14) (see Table 1). On a scale ranging from 0 to 27, the average total depressive symptom severity score was 3.39 (SD = 4.35). Approximately 18 percent (18.2%, n = 344) reported mild depressive symptoms (scores ranging from 5 to < 10) and 8.9% (n = 168) scored in the moderate to severe depressive symptom level range (scores ≥ 10). The average score for individual-level and community-level collective efficacy was 36.10 (SD = 7.64) and 36.12 (SD = 4.29), respectively. After accounting for missing data across the predictor variables, 1893 cases remained for all analyses below.

**Table 1:** Means or proportions with standard deviations for participant characteristics (n = 1893).

Dependent and independent variables		Mean or % (SD)
Collective efficacy	Individual-level collective efficacy^1^	36.10 (7.64)
	Community-level collective efficacy^1^	36.12 (4.29)
Depressive symptoms	Depressive symptom severity score^1^	3.39 (4.35)
Demographics/individual factors	Female^2^	0.82 (0.39)
	Age^1^	47.52 (10.31)
	White^2^	0.73 (0.44)
	College or more^2^	0.50 (0.50)
	Married^2^	0.66 (0.47)
	Injury/damage^2^	0.58 (0.49)
Community factors	Community storm damage^1^	1.50 (1.14)

^1^ Mean

^2^%

### Multilevel Analyses

#### Depressive symptom severity

Two random coefficient effects analyses were conducted to evaluate the associations between a) individual-level collective efficacy and depressive symptom severity and b) community-level collective efficacy and depressive symptom severity. These relationships were considered while adjusting for the aforementioned individual and community sociodemographic variables, individual injury/damage, community storm damage, the interaction between injury/damage and collective efficacy, and the interaction between injury/damage and community storm damage.

#### Individual-level collective efficacy

Beginning with a model containing all covariates, analyses revealed that a higher level of individual-level collective efficacy was associated with a significantly lower score of depressive symptom severity. In addition, having high injury/damage was positively related to depressive symptom severity, other variables being equal. Further, the interaction between individual injury/damage and community storm damage was not significant (95% CI: -0.06, 0.82). We also examined the model after removing the nonsignificant two interactions and three socioeconomic characteristics. This modification to the model did not significantly change the model chi-square and the parameter estimates remained essentially unchanged. We used the full model because theoretical as well as previous empirical studies suggest the relevance of these variables to both depression and collective efficacy and similar constructs [13]. In the selected full model, after adjusting for all other covariates, a one point increase in individual-level collective efficacy for those with low injury/damage was associated with a depressive symptom severity score that was 0.09 points lower (95% CI: -0.12, -0.06). On the other hand, for those with high injury/damage, a one point increase in individual-level collective efficacy resulted in a depressive symptom severity score that was 0.13 points lower (-0.09–0.04), other variables being equal (the score difference in this group was not statistically significant: see Table 2). The intra-communities correlation for the individual-level efficacy model was 0.038 (Model χ^2^ = 89.10, p < 0.001).

**Table 2:** Parameter estimates of two multilevel linear regression models on Depressive Symptom Severity (n = 1893).

		Individual-level collective efficacy models	Community-level collective efficacy models
Variable		Full model b (95% confidence interval)	Full model b (95% confidence interval)
Fixed effect	Intercept	12.35** (12.14, 12.56)	12.41** (12.20, 12.61)
	Collective efficacy	-0.09** (-0.12, -0.06)	-0.09** (-0.13, -0.04)
	Sex	0.35 (-0.26, 0.95)	0.28 (-0.34, 0.90)
	Age	-0.02 (-0.04, 0.01)	-0.02* (-0.04, -0.00)
	Race/ethnicity	0.92** 0.38, 1.45)	1.03** (0.48, 1.58)
	Education	-0.32 (-0.76, 0.13)	-0.31 (-0.76, 0.14)
	Marital status	-0.55* (-1.00, -0.10)	-0.70** (-1.15, -0.25)
	Injury/damage	1.16** (0.66, 1.66)	1.20** (0.67, 1.72)
	Community storm damage	0.09–0.09, 0.27)	0.05 (-0.14, 0.24)
	Concentrated disadvantage	0.07 (-0.16, 0.30)	0.11 (-0.12, 0.34)
	Immigrant concentration	-0.08 (-0.31, 0.16)	-0.15 (-0.39, 0.09)
	Residential stability	0.00 (-0.23, 0.24)	0.03(-0.19, 0.26)
	Collective efficacy x injury	-0.04 (-0.11, 0.03)	-0.03 (-0.16, 0.11)
	Storm x injury	0.38 (-0.06, 0.82)	0.35 (-0.11, 0.80)
Random effect	Between communities (τ00)	0.67* (0.08, 1.27)	0.63* (0.00, 1.26)
	Slope of col eff^a^/injury^b^ (τ11)	0.01** (0.00, 0.02)	1.15 (-1.13, 3.42)
	Between intercept & slope (τ10)	-0.10** (-0.15, 0.05)	1.44** (0.49, 2.38)
	Within communities (σ^2^)	16.90** (15.68, 18.11)	17.70** (16.43, 18.97)
	ICC	0.038	0.034
Model χ^2^		89.10**	43.80**

*p < 0·05;

**p < 0·01

#### Community-level collective efficacy

In a model with all covariates included, analyses revealed that a higher level of community-level collective efficacy was associated with a significantly lower score of depressive symptom severity. Further, having high injury/damage was associated with a higher level of depressive symptom severity. We examined the model after removing the nonsignificant two interactions and three socioeconomic characteristics. Making this change to the model did not significantly change the model chi-square and the parameter estimates remained essentially unchanged. Again, we selected the full model because theoretical and previous empirical studies suggest the relevance of these variables to depression and collective efficacy or similar constructs [18]. In the full model, including all covariates, a one point increase in community-level collective efficacy for those with low injury/damage was also associated with a depressive symptom severity score that was 0.09 points lower, though with a slightly different 95% confidence interval (95% CI: -0.13, -0.04). For those with high injury/damage, a one point increase in individual-level collective efficacy resulted in a depressive symptom severity score that was 0.12 points lower (-0.09–0.03), other covariates being equal (again the score difference in this group was not statistically significant; see Table 2). The intra-communities correlation for the community-level efficacy model was 0.034 (Model χ^2^ = 43.80, p < 0.001). To assess the impact the small average sample size might generate, we performed an analysis combining the zip codes with small sample sizes with those having a sufficient number of participants. Specifically, we used the propensity score matching approach to merge the subjects residing in zip codes with less than five participants into the zip codes of larger sample sizes that had the closest propensity score. In this matching process, the propensity is specified as the probability of low zip-specific collective efficacy (cut-point is 33), using 14 zip-level variables as predictors. The merging resulted in 135 communities. Using this smaller number of communities did not change the analytic results significantly, and therefore, the original, actual zip code was used in formal analyses. Technically, low reliability in some level-2 units was adequately handled by the application of the empirical Bayesian approach available in the SAS PROC MIXED procedure [33, 34].

## Discussion

Community collective efficacy is associated with mental health and in particular, depression [18, 19], including following a disaster. Most studies assess community resources or characteristics at the individual level, and do not address the influence of community-level collective efficacy. This study examined the relationship of depression and collective efficacy at both the individual level (the perception of collective efficacy) and the community level using zip codes to define the community units. We found that both higher community-level and individual- level collective efficacy were associated with lower depressive symptoms. This similarity may reflect neighborhoods that are cohesive in collective efficacy, i.e., individual perceptions are similar within neighborhoods and therefore may be another indicator of a cohesive community. Future studies should examine this. Alternatively, those who are depressed are more likely to report lower perceived collective efficacy and to live in the same community. The relatively small number of individuals in some of our zip codes may have also influenced this finding.

Among those individuals who had low levels of injury/damage, higher community-level collective efficacy was associated with lower depressive symptoms, even after adjusting for individual sociodemographics, community socioeconomic characteristics, the individuals’ degree of injury/damage, and the community level of storm damage. Among those with high individual injury/damage, higher community-level collective efficacy was also associated with a lower depressive symptom score, a relatively though not statistically higher effect compared to those with low injury/damage. These reductions were also true for individual-level (perceived) collective efficacy. These findings support a possible role of collective efficacy in depression in post disaster communities, perhaps more so in communities with lower injury/damage, suggesting a ceiling effect of injury/damage above which collective efficacy may not be associated with depression.

Though fairly minor at face value, such statistically meaningful decreases in symptoms are important to understanding mechanisms in population health and psychiatric symptoms. Although we cannot infer causality, and in particular, reverse causality may be possible, these changes may imply a considerable alleviation of distress when even such a small change is applied to a large population after a disaster. Our recent study of PTSD and collective efficacy showed a similar pattern in which the effects of collective efficacy were fairly low but they translated into a decreasing trend in PTSD when a wide range of collective efficacy is considered [8]. The regression coefficients of collective efficacy on depressive symptoms are lower than those reported for PTSD symptom scores (in the case of community level, -0.09 versus -0.17). However, this reflects an effect on a health score ranging from 0 to 27 for depression compared to a range from 17 to 85 for PTSD. Therefore, a one-unit decrease in depressive symptoms is more meaningful than the same decrease for PTSD, as the score varies within a much narrower score interval. Therefore, future studies should examine the possibility that community collective efficacy may actually have a stronger impact on depressive symptoms than on PTSD.

As mentioned earlier, due to the cross-sectional nature of this study, assumptions about causal direction are challenging. If perceived collective efficacy is causally related to depression it may be that higher perceived collective efficacy is protective for depression, and lower collective efficacy leads to higher depression. Alternatively, it may be that depression makes one perceive collective efficacy in a more negative light, suggesting further research is needed to better understand the relationship between depression and perceived collective efficacy.

The present findings must be interpreted in terms of several methodological considerations. Since this is a cross-sectional study, further research using longitudinal designs is needed. Because the sample was subdivided into zip codes, the sample size may affect the representativeness of the zip code. While this is a reasonable choice, it is plausible that in some cases, zip codes cross neighborhoods. It should be noted that this study examined general depression that was not necessarily related to trauma and possibly had an onset that preceded exposure to the hurricanes. Although the current injury/damage assessment captured this variable adequately, future studies should include general items regarding damage to property.

Although assumptions about the causal nature of our findings cannot be assumed, our study highlights the possible importance of programs that enrich community collective efficacy that can be integrated into public health disaster planning. Involvement of community members in disaster planning can foster community cohesion and a sense of working together in the face of adversity. This is particularly important for public health care workers who have a responsibility to care for community members while simultaneously managing their own responses to a disaster. Community-level interventions should make resources available across members of the community, strengthening social cohesion and individual and collective response to disaster events. Opportunities to study such interventions will clarify the causal relations, and inform programs that promote and strengthen individual and community resources and mental health in public health workers.
